# Natural Tolerance to Ischemia and Hypoxemia in Diving Mammals: A Review

**DOI:** 10.3389/fphys.2019.01199

**Published:** 2019-09-20

**Authors:** Kaitlin N. Allen, José Pablo Vázquez-Medina

**Affiliations:** Department of Integrative Biology, University of California Berkeley, Berkeley, CA, United States

**Keywords:** hypoxia, cetacean, pinniped, oxidative stress, inflammation

## Abstract

Reperfusion injury follows ischemia/reperfusion events occurring during myocardial infarction, stroke, embolism, and other peripheral vascular diseases. Decreased blood flow and reduced oxygen tension during ischemic episodes activate cellular pathways that upregulate pro-inflammatory signaling and promote oxidant generation. Reperfusion after ischemia recruits inflammatory cells to the vascular wall, further exacerbating oxidant production and ultimately resulting in cell death, tissue injury, and organ dysfunction. Diving mammals tolerate repetitive episodes of peripheral ischemia/reperfusion as part of the cardiovascular adjustments supporting long duration dives. These adjustments allow marine mammals to optimize the use of their body oxygen stores while diving but can result in selectively reduced perfusion to peripheral tissues. Remarkably, diving mammals show no apparent detrimental effects associated with these ischemia/reperfusion events. Here, we review the current knowledge regarding the strategies marine mammals use to suppress inflammation and cope with oxidant generation potentially derived from diving-induced ischemia/reperfusion.

## Introduction

The ability to manage body oxygen stores in the face of environmental hypoxia constrains the life history of many vertebrate taxa. In humans, oxygen management is critically important in clinical settings where both acute and chronic conditions such as organ transplantation and intermittent hypoxia contribute to ischemic injuries. Diving mammals, however, experience fluctuations in blood flow and oxygen saturation without sustaining such injuries. A range of physiological mechanisms for coping with finite oxygen availability has been identified to date (for recent comprehensive reviews on the topic, see Davis, 2014; Blix, 2018; Ponganis, 2019). The first – and perhaps most straightforward – of these mechanisms is increased mass-specific body oxygen stores which delay the onset of hypoxemia and tissue hypoxia and prolong submergence times (Ponganis et al., 1993; Kanatous et al., 1999, 2002; Burns et al., 2007). Similarly, splenic contraction increases circulating oxygen levels during apnea in pinnipeds (Castellini and Castellini, 1993; Elsner, 1995; Hurford et al., 1996; Thornton et al., 2001). Populations of human breath-hold divers also demonstrate enlarged spleens and positive selection of genes implicated in spleen size (PDE10A) and regulation of vasomotor tone (BDKRB2) (Hurford et al., 1990; Ilardo et al., 2018). In marine mammals these “onboard” oxygen stores are likely sufficient to support normal aerobic function in peripheral organs including the liver and kidneys during most dives (Davis et al., 1983). Additionally, the majority of pinniped dives occur within the aerobic dive limit (Kooyman et al., 1983; Davis and Kanatous, 1999; Davis, 2014). However, diving mammals experience hypoxemia during routine breath holding within aerobic limits, tolerating lower arterial and venous oxygen saturations than most terrestrial mammals including humans (Ferretti et al., 1991; Stockard et al., 2007; Ponganis et al., 2008; Lindholm and Lundgren, 2009; Meir et al., 2009, 2013; McDonald and Ponganis, 2013; Tift et al., 2018). The specific molecular and cellular pathways that protect marine mammals from injuries driven by fluctuations in blood flow and local oxygen tensions remain largely unexplored.

**Figure 1:**
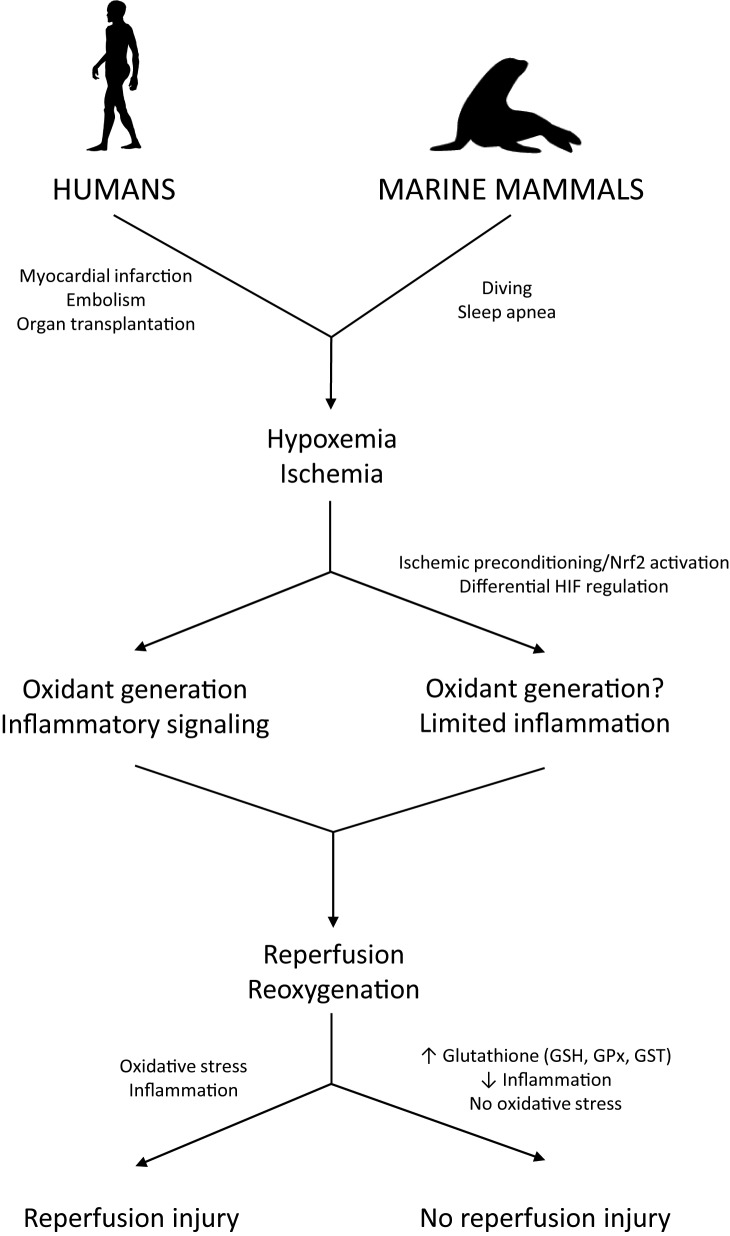
Biochemical mechanisms leading to the prevention of reperfusion injury in diving mammals. Ischemia/reperfusion events are associated with oxidative stress and inflammation in humans but are well tolerated by diving mammals. The mechanisms marine mammals use to prevent inflammation and oxidative stress derived from diving-induced ischemia and hypoxemia are still under investigation but likely include upregulation of genes involved in antioxidant defense and hypoxia tolerance *via* preconditioning-like responses that involve activation of the transcription factors Nrf2 and HIF-1.

In humans, hypoxemia induces cell death and tissue injury *via* inflammatory, necrotic, and apoptotic pathways (Gottlieb et al., 1994; Saikumar et al., 1998; Li and Jackson, 2002; Sendoel and Hengartner, 2014). Limited oxygen availability during hypoxia impairs mitochondrial respiration, leading to a drop in intracellular ATP levels. Reoxygenation after hypoxia increases oxidant generation from enzymatic systems (e.g., xanthine oxidase, NADPH oxidases) and mitochondria, carrying an additional threat of oxidative injury to cells and tissues and potentially compromising organismal health and survival (Figure 1; McCord, 1985; Kalogeris et al., 2012). Reperfusion injuries are well documented in humans, particularly with respect to myocardial infarction, ischemic stroke, and organ transplantation (Yellon and Hausenloy, 2007; Iadecola and Anrather, 2011; Salvadori et al., 2015). Thus, both hypoxemia and reduced peripheral perfusion associated with diving can potentially contribute to inflammation and oxidative stress in diving mammals. Remarkably, diving mammals appear to tolerate such conditions without injury. Therefore, understanding the mechanisms underlying this tolerance may yield insight into translational applications for human health.

## The Mammalian Diving Response, Ischemia, and Hypoxia

The mammalian diving response consists of several coordinated physiological adjustments originally considered to protect the hypoxia-sensitive central nervous system while oxygen availability is limited during a dive (Irving, 1938; Irving et al., 1941, 1942; Bron et al., 1966). More recently, it was discovered that the diving response also maximizes the aerobic dive limit without compromising central nervous system function (Davis and Kanatous, 1999; Davis, 2014). The three primary components of the diving response are apnea, bradycardia, and peripheral vasoconstriction; all three have been studied in multiple marine mammal species using different experimental approaches (Elsner, 1999; Davis and Williams, 2012; Williams et al., 2015; Blix, 2018; Ponganis, 2019). In pinnipeds, apnea alone is sufficient to induce both bradycardia and vasoconstriction independent of whether the animal is diving (Andrews et al., 1997; Ponganis et al., 2008); bradycardia is further modulated by water temperature, extent of facial submersion, cognition (i.e., anticipation) and exercise (Ridgway et al., 1975; Davis and Kanatous, 1999; Davis and Williams, 2012; Williams et al., 2015; Elmegaard et al., 2016; Kaczmarek et al., 2018). Peripheral perfusion and vasoconstriction have historically been difficult to measure in marine mammals. Early forced submersion studies in Weddell seals indicated an extensive, profound, near-cessation of blood flow to peripheral tissues including the kidney, liver, and spleen during forced dives (Zapol et al., 1979). Similarly, comparative studies showed that seal kidneys exposed to *ex vivo* ischemia recovered both blood flow and urine production upon reperfusion, while dog kidneys did not (Halasz et al., 1974). The magnitude of the physiological response occurring in free dives is likely different from that observed during forced submersions (Hill et al., 1987; reviewed in Ponganis et al., 2011). Measurements of peripheral perfusion during voluntary apneas, however, have focused on skeletal muscle rather than splanchnic organs (Guyton et al., 1995; Ponganis et al., 2008); reduced muscle perfusion during sleep apneas and diving allows for utilization of myoglobin-bound oxygen by the muscle (Qvist et al., 1981; Guyton et al., 1995; Meir et al., 2009; Wright and Davis, 2015). A lack of instrumentation has hampered direct observations of splanchnic organ perfusion during free dives. Recently, an overall reduction in blubber hemoglobin concentration and saturation was observed in captive harbor seals voluntarily diving within aerobic limits (McKnight et al., 2019), suggesting reduced perfusion to the periphery during routine dives. Despite reduced cardiac output and peripheral oxygen consumption during diving, however, marine mammals deplete central oxygen stores during routine voluntary dives (Shaffer et al., 1997; Williams et al., 1999; Meir et al., 2009, 2013; McDonald and Ponganis, 2013; Tift et al., 2018). As a result, even continuously perfused tissues such as the brain likely experience reductions in oxygen tension as a result of diving-induced hypoxemia (Elsner et al., 1970; Kerem and Elsner, 1973; McKnight et al., 2019). Elevated neuroglobin levels and selective brain cooling may supplement cerebral oxygen stores while decreasing demand during diving (Odden et al., 1999; Williams et al., 2008; Blix et al., 2010; Schneuer et al., 2012). Moreover, the seal brain is capable of both producing and consuming lactate *in vivo* (Murphy et al., 1980), and glucose deprivation during hypoxia does not appear to negatively impact neuronal function in seal brain slices, suggesting that the seal brain may tolerate these insults (Czech-Damal et al., 2014; Geiseler et al., 2016). Recent transcriptomic analyses suggest that metabolic shifts and upregulation of several major stress response pathways also help protect the seal brain against hypoxic injury (Fabrizius et al., 2016; Hoff et al., 2017). Thus, understanding the mechanisms that protect the marine mammal brain during diving-induced ischemia/reperfusion can potentially reveal new targets for pharmacological interventions in human brain injury and stroke patients.

## Consequences of Hypoxia and Ischemia: Oxidant Generation, Inflammation, and Oxidative Stress

The complex mechanisms of ischemic tissue damage have been studied extensively in biomedical models due to their clinical relevance to human conditions including organ transplantation, myocardial infarction, and stroke. Oxygen deprivation during ischemia depletes intracellular ATP; subsequently, ATP degradation products including xanthine and hypoxanthine accumulate (McCord, 1985). Ischemia also dysregulates calcium levels, activating calcium-dependent proteases which cleave xanthine dehydrogenase to generate the active form of the enzyme, xanthine oxidase (Arnould et al., 1992; Berna et al., 2002). Increases in intracellular calcium during ischemic events also induce the activation of NADPH oxidases and promote mitochondrial superoxide radical generation (Brookes et al., 2004; Granger and Kvietys, 2015). Upon reperfusion/reoxygenation, xanthine and hypoxanthine are oxidized by xanthine oxidase, generating superoxide radical and hydrogen peroxide (Chambers et al., 1985; McCord, 1985; Granger, 1988). Similarly, superoxide is generated by reverse electron transport at the mitochondrial complex I during reperfusion (Chouchani et al., 2015). In the vascular endothelium, superoxide generated by NADPH oxidases activated *via* mechanosignaling during ischemic alterations in shear stress contributes to tissue injury *via* the formation of peroxynitrite (Beckman et al., 1990; Al-Mehdi et al., 1998; Fisher et al., 2001; Noel et al., 2013; Browning et al., 2014). Furthermore, inflammatory molecules generated or activated by endothelial NADPH oxidase-mediated redox signaling (e.g., CAMs, selectins, NF-κB, and NLRP3 inflammasomes) prime the vasculature for neutrophil adherence and infiltration, promoting further tissue injury during reperfusion (Eltzschig and Eckle, 2011; Iadecola and Anrather, 2011). In diving seals, convective oxygen transport to peripheral tissues remains sufficient to support aerobic metabolism during most dives (Davis et al., 1983; Davis and Kanatous, 1999); however, routine hypoxemia coupled with reoxygenation upon surfacing (Qvist et al., 1986; Meir et al., 2009, 2013; McDonald and Ponganis, 2013; Tift et al., 2018), along with potential alterations in blood flow derived from peripheral vasoconstriction, expose the vascular endothelium to frequent fluctuations in shear and oxygen tension that resemble pathological ischemia/reperfusion events in humans.

Marine mammals do not sustain the reperfusion injuries associated with hypoxemia and ischemia/reperfusion events in humans; however, the mechanisms regulating this oxidant balance in marine mammals remain unclear. Increased purine recycling has been proposed as a mechanism to limit xanthine oxidase-derived oxidant generation in marine mammals (López-Cruz et al., 2016), but support for this hypothesis is mixed (Soñanez-Organis et al., 2012; del Castillo Velasco-Martínez et al., 2016). Early work revealed that seal tissues accumulate hypoxanthine after simulated ischemia and are capable of generating oxidants *ex vivo* (Elsner et al., 1995, 1998; Zenteno-Savín et al., 2002). Similarly, circulating concentrations of xanthine and hypoxanthine increase during spontaneous on-land sleep apneas in elephant seals (Vázquez-Medina et al., 2011d). Moreover, tissue capacity to generate oxidants increases with postnatal maturation in hooded seal skeletal muscle (Vázquez-Medina et al., 2011a), while circulating and muscle levels of xanthine oxidase increase after repetitive sleep apneas in elephant seals (Vázquez-Medina et al., 2011d). In our preliminary observations, we have found that seal endothelial cells in primary culture generate oxidants following exposure to hypoxia/reoxygenation and when incubated with known activators of NADPH oxidases (Vázquez-Medina et al., 2018). Together, these results suggest that avoiding oxidant generation is not a strategy seals use to cope with diving-induced ischemia/reperfusion. Of note is recent evidence showing alterations in the nitric oxide-soluble guanylyl cyclase-cGMP (NO-cGMP) pathway in peripheral tissues of Weddell seals compared to non-diving vertebrates (Hindle et al., 2019) and the previously observed absence of nitric oxide in the exhalate of Weddell seals after voluntary dives (Falke et al., 2008). Such alterations in the NO-cGMP pathway could help maintain differential perfusion during a dive (Hindle et al., 2019) while preventing the formation of peroxynitrite *via* the reaction of NO with superoxide generated in response to diving-induced ischemia/reperfusion (Ischiropoulos et al., 1992; Radi et al., 2001).

Besides their proven role in cell and tissue injury, oxidants participate in essential physiological functions, including host defense and neovascularization (Babior, 1999; Tojo et al., 2005; Browning et al., 2014). Similarly, at sub-toxic levels, oxidants such as hydrogen peroxide, which can be generated directly by xanthine oxidase and certain NADPH oxidases or by dismutation of superoxide, mediate a plethora of redox-dependent pathways related to calcium signaling, protein phosphorylation, and transcription factor activation (Suzuki et al., 1997). Transcription of most antioxidant genes is under control of the nuclear factor E2-related factor 2 (Nrf2), which is activated in response to temporal increases in intracellular oxidants or other electrophiles (Itoh et al., 1997, 1999). Stimulation of Nrf2 by the lipid peroxidation product 4-hydroxy-2-nonenal (4-HNE) is involved in neuro- and cardio-protection against oxidative stress after ischemic preconditioning (Calvert et al., 2009; Zhang et al., 2010; Bell et al., 2011). In humans, ischemic preconditioning reduces the risk of myocardial injury after coronary artery bypass graft surgery (Thielmann et al., 2013). In elephant seals, repetitive spontaneous sleep apneas result in blood oxygen depletion, reduced muscle blood flow, decreased tissue P_O2_, and increased 4-HNE, nuclear Nrf2 levels, and antioxidant enzyme expression in skeletal muscle (Ponganis et al., 2002, 2006, 2008; Stockard et al., 2007; Vázquez-Medina et al., 2011d, 2012). These results suggest that repetitive breath holding in marine mammals resembles preconditioning responses that protect tissues from oxidant generation during diving.

## Coping With Diving-Induced Hypoxia and Ischemia: Counteracting Oxidative Stress and Preventing Inflammation

High activity and expression of antioxidant enzymes, particularly those related to the glutathione system, have been observed across diving birds and mammals (Murphy and Hochachka, 1981; Corsolini et al., 2001; Wilhelm Filho et al., 2002; Vázquez-Medina et al., 2006, 2007; Zenteno-Savin et al., 2010; García-Castañeda et al., 2017). Baseline circulating and tissue antioxidant levels are higher in diving versus non-diving birds and mammals, supporting the hypothesis that a robust antioxidant defense system mitigates injury from diving-induced oxidant generation in marine vertebrates (Corsolini et al., 2001; Wilhelm Filho et al., 2002; Vázquez-Medina et al., 2006, 2007, 2012; Zenteno-Savin et al., 2010, 2011; García-Castañeda et al., 2017). Whether this relationship between antioxidant levels and diving capacity holds across diving species remains unclear; interspecies comparisons of diving capacity and antioxidant levels are difficult to isolate from confounding species-specific life history factors such as fasting and maturation (Cantú-Medellín et al., 2011; Righetti et al., 2014; Colominas-Ciuró et al., 2017; García-Castañeda et al., 2017). However, in phocid seals, the antioxidant system develops alongside diving capacity during postnatal maturation and does not decline with aging, suggesting a link between diving ability and antioxidant defenses (Vázquez-Medina et al., 2011b,c; Allen et al., 2019).

Despite strong antioxidant responses, lipid peroxidation has been detected in marine mammal tissues under basal conditions, during aging, and after repetitive apneas (Zenteno-Savín et al., 2002; Vázquez-Medina et al., 2011d, 2012; Allen et al., 2019). Changes in lipid peroxidation levels after oxidant-generating challenges, however, are limited in contrast to what is observed in non-hypoxia tolerant mammals undergoing similar fluctuations in blood flow and tissue oxygenation (Figure 1; Sakamoto et al., 1991; Szabó, 1996; Paradies et al., 1999; Zenteno-Savín et al., 2002; Vázquez-Medina et al., 2007, 2011d; Kalogeris et al., 2012). Consequently, observed lipid peroxidation levels may be within tolerable limits for marine mammals. As discussed above, antioxidant gene expression in marine mammals is likely regulated by redox signaling derived from repetitive apneic periods. Accordingly, sub-lethal levels of oxidants and other potent electrophiles such as lipid peroxidation products (e.g., 4-HNE) may modulate antioxidant gene transcription, contributing to the protective “preconditioning” effect of repeated diving (Zhang et al., 2010; Wang et al., 2018).

In concert with oxidant generation, ischemic inflammation also contributes to reperfusion injury (McCord and Roy, 1982; McCord, 1987; Eltzschig and Carmeliet, 2011; Iadecola and Anrather, 2011). A limited body of recent work has started to address inflammatory responses in diving mammals. Serum from deep-diving seals protected both seal and mouse cells against LPS-induced inflammation *in vitro*, suggesting an as-yet-undetermined anti-inflammatory component in circulation (Bagchi et al., 2018). Similarly, elevated levels of carboxyhemoglobin may protect against inflammatory injury during reperfusion in marine mammals despite detracting from the overall oxygen-binding capacity of the blood (Otterbein et al., 2000; Ozaki et al., 2012; Tift et al., 2014). Thus, diving mammals appear to utilize both anti-inflammatory and antioxidant strategies to mitigate tissue damage potentially derived from diving-induced hypoxemia and ischemia/reperfusion, though the molecular and biochemical bases of this control remain unknown.

Of note is evidence showing that prolonged food deprivation does not increase systemic inflammation but does result in increased muscle TNFα mRNA and protein levels in elephant seals (Vázquez-Medina et al., 2010; Suzuki et al., 2013). Similarly, breeding haul-outs are associated with systemic inflammatory responses in elephant seals, and plasma haptoglobin levels are increased in declining and nutritionally stressed populations of harbor seals and Steller sea lions (Zenteno-Savín et al., 1997; Peck et al., 2016). These results suggest that, rather than being blunted, inflammatory responses in seals are tightly regulated at both systemic and tissue levels and that modulation of these processes may contribute significantly to avoiding diving-induced inflammation. In support of this idea, endocrine manipulation (ACTH stimulation) studies coupled with transcriptomic analyses in elephant seals show suppression of the NF-κB pathway in seal muscle (Khudyakov et al., 2015). Our unpublished observations also suggest regulation of systemic inflammatory components (C-reactive protein levels) in response to both ACTH stimulation and local (muscle) blockade of the glucocorticoid receptor in elephant seals.

## Molecular Underpinnings of Hypoxia and Oxidative Stress Tolerance in Marine Mammals

Recent genetic and molecular work has begun to address the underlying gene-level modifications contributing to the physiological adjustments supporting diving in marine mammals. The hypoxia-inducible factor 1 (HIF-1) is considered the master regulator of the molecular response to hypoxia across taxa (Soitamo et al., 2001; Nikinmaa et al., 2004; Semenza, 2008; Weidemann and Johnson, 2008). Functional HIF-1 is composed of two subunits, HIF-1α and HIF-1β. Under normoxic conditions, the pVHL-ubiquitin-proteasome proteolytic pathway continuously degrades HIF-1α. During hypoxia, this degradation is halted and HIF-1α dimerizes with HIF-1β, translocating into the nucleus where it regulates transcription of genes involved in angiogenesis, erythropoiesis, and proliferation (Wang et al., 1995; Lee et al., 2004). Convergent substitutions in the HIF-1α amino acid sequence across hypoxia-tolerant mammals including cetaceans, high altitude ungulates, and subterranean rodents suggest a critical role for HIF-1α regulation in natural hypoxia tolerance (Zhu et al., 2018). Seals possess a single copy of the HIF-1α gene; it is similar in sequence to terrestrial mammal HIF-1α, though with several amino acid differences in the oxygen-dependent degradation domain (Johnson et al., 2005). Seal tissues with higher HIF-1α protein levels show less overall protein oxidation, suggesting that HIF-1α expression protects against oxidative stress in marine mammals (Johnson et al., 2004). Interestingly, amino acid sequence differences in cetacean HIF-1α likely affect HIF-1α sensitivity and responsiveness to changing oxygen conditions rather than establishing a constitutively active response (Bi et al., 2015). In our *in vivo* experiments we have observed marked HIF-1α upregulation in elephant seal muscle in response to prolonged fasting and repetitive sleep apneas (Vázquez-Medina et al., 2011d; Soñanez-Organis et al., 2014). Moreover, our preliminary observations suggest that HIF-1α stabilization is rapid and sustained in response to hypoxia in seal endothelial cells in primary culture in comparison to the response observed in human cells. Together, these studies suggest a critical role of HIF-1α in mediating hypoxia tolerance in marine mammals.

Recent phylogenomic studies have begun to uncover additional molecular mechanisms underpinning ischemia/reperfusion tolerance in marine mammals, including the expansion and positive selection of several gene families related to oxidative stress tolerance and oxygen management. Most work has considered cetacean species; pinniped genomes have generally been less available. In strong agreement with the current physiological understanding of antioxidants in both cetaceans and pinnipeds, several genes in the glutathione system – including glutathione reductase, glutathione peroxidases, and γ-glutamylcysteine ligase – are expanded, under positive selection, and/or have amino acid changes in cetaceans (Yim et al., 2014; Zhou et al., 2018). Two peroxiredoxin gene families (PRDX1 and PRDX3) are also expanded in cetacean lineages (Yim et al., 2014; Zhou et al., 2018), suggesting an augmented capacity for redox signaling and antioxidant protection (Perkins et al., 2015). Moreover, an inactivating mutation in the cetacean gene encoding Polμ, a polymerase with low fidelity in repairing oxidative DNA damage, suggests that reliance on a higher fidelity polymerase (Polλ) may confer tolerance to oxidative damage (Pryor et al., 2015; Huelsmann et al., 2019). In cetaceans, contracted gene families involved in the acute inflammatory response and repair of lipid peroxidation support physiological data in deep-diving pinnipeds that suggest that these animals may have evolved mechanisms to cope with ischemic inflammation associated with diving (Tift et al., 2014; Bagchi et al., 2018; Meyer et al., 2018; Zhou et al., 2018). Positive, convergent selection for a gene encoding a lung surfactant protein (SFTPB) in cetaceans, pinnipeds, and sirenians could help explain the rapid distribution of pulmonary surfactant necessary to sustain and tolerate repeated lung collapse and re-inflation in diving mammals (Miller et al., 2004, 2006; Spragg et al., 2004; Gutierrez et al., 2015; Chikina et al., 2016).

Managing body oxygen stores and tissue oxygen supply while diving is of paramount importance for marine mammals. Hemoglobin and myoglobin are central to this process; both are under positive selection in cetaceans (Tian et al., 2016). In the case of myoglobin, an augmented net surface charge observed across all diving mammals might allow for high, functional muscle myoglobin concentrations, thereby increasing body oxygen stores (Mirceta et al., 2013). Several metabolic gene families are also under positive selection in cetaceans, including TCA cycle enzymes citrate synthase and pyruvate carboxylase (Tian et al., 2017). Cetacean-specific amino acid changes in and expansion of lactate dehydrogenase and monocarboxylate transporter 1 genes suggest an increased ability to metabolize lactate after dives exceeding aerobic limits (Yim et al., 2014; Tian et al., 2017). Lactate has been increasingly recognized as a primary metabolic fuel rather than a waste product (Hui et al., 2017; Brooks, 2018). Therefore, these observations could help explain why several marine mammals routinely dive beyond their calculated aerobic dive limits and can spend up to 90% of their time at sea submerged with minimal recovery periods (Le Boeuf et al., 1988; Costa et al., 2001; Butler, 2006; Robinson et al., 2012; Meir et al., 2013).

## Conclusions and Future Directions

Marine mammals experience diving-induced hypoxemia and ischemia/reperfusion events without apparent detrimental effects. Physiological, biochemical, and genomic studies have begun to uncover the mechanisms underlying such extreme tolerance. Among those mechanisms, coordinated body-wide responses to delay the onset of tissue hypoxia, counteract oxidant generation and prevent inflammation are critical. Convergent genomic changes across marine mammal lineages hint at the evolutionary underpinnings of the physiological adaptations supporting mammalian diving (Chikina et al., 2016). The increasing availability of genome sequences from additional species will certainly strengthen these studies. Functional studies dissecting the cellular and molecular underpinnings that confer tolerance to hypoxia and ischemia in marine mammals are yet to be conducted. We and others are currently carrying out experiments using *ex vivo* systems that are amenable to physiological manipulation and molecular perturbation in an effort to provide the missing link between genomic- and organismal-level investigations. Identifying the drivers of ischemic and hypoxemic tolerance in marine mammals can provide a mechanistic understanding of natural tolerance to such conditions while aiding in translation to clinical applications.

## Author Contributions

KA and JV-M wrote and edited the manuscript.

### Conflict of Interest

The authors declare that the research was conducted in the absence of any commercial or financial relationships that could be construed as a potential conflict of interest.
